# Subcutaneous Fibrous Nodules

**Published:** 1904-10-22

**Authors:** 


### Hospital Clinics.

### SUBCUTANEOUS FIBROUS NODULES.

The recognition of subcutaneous fibrous nodules as a manifestation of rheumatism in childhood was without question a distinct clinical advance. It may be described as one of a series of events by which the outlook of professional opinion in relation to rheumatism in early life was extended and enlarged. There was, or rather at first there appeared to be, this further advantage attached to these nodules, namely, that they offered themselves as conclusive and decisive evidence of rheumatism. This was all the more welcome, seeing that many of the phenomena newly brought within the area of the rheumatic claim had, in the individual patient, a decidedly ambiguous significance. For even the strongest advocate of the doctrine that such conditions as tonsillitis, pleurisy, erythema, etc., must be included within the rheumatic series will hardly urge that these events never occur apart from rheumatism. Hence in any given case the occurrence of any one of these conditions sounds only an uncertain note. But with the eruption of the subcutaneous nodule, it was said, all uncertainty ceases. Whatever doubt may cling to other symptoms none remains here—the subcutaneous fibrous nodule is solely and purely rheumatic in origin, and its appearance decides the diagnosis. Now it must be at once admitted that the broad position that the development of subcutaneous fibrous nodules is a not infrequent and an extremely valuable evidence of rheumatism in early life remains unchallenged. The careful and exact description of these nodules as given by Barlow and Warner in 1881 has undergone no substantial modification. On the contrary, it may still be recognised as not only the truth but almost, at least, the whole truth. In the enormous majority of instances the appearances these authorities described occur in rheumatic children, and in practical clinical work there is hardly room for doubt and uncertainty as to the interpretation to be attached to them. Small, firm nodules developing under the skin, more especially over bony prominences such as the fingers, elbows, spines of the vertebræ, etc., often appearing rapidly and lasting but for a period of days or weeks, are now allowed by every observer to be amongst the most distinctive results of rheumatic disease. That is to say, that in whatever respect the original position has been modified, its practical application in the field of diagnosis stands secure. In some few instances, more or less doubtful exceptions have been recorded, and on the point of doctrinal teaching it must be admitted that a considerable body of evidence has been produced with a view to challenge the proposition that subcutaneous fibrous nodule formation never occurs apart from the rheumatic process. All this has its own interest and value, and note may be taken of it even whilst allowing that it hardly affects the ordinary field of clinical work. One of the most definite of the exceptions above alluded to is a case recorded by Dr. George Carpenter.[1] The imitation in this instance of the so called rheumatic nodules was most close, as may be seen in the drawing which accompanies Dr. Carpenter's article. Yet, though the child was watched for more than two years, no evidences of rheumatism appeared during that period, and there was no suggestion of rheumatism in the family history. Other less marked cases of the same order have been recorded, so that one must admit that even in their most typical form, and in young patients, subcutaneous fibrous nodules may appear without other evidence of rheumatism, and possibly therefore entirely apart from rheumatic disease. Next, as bearing on the general theoretical position, comes the question of the occurrence of subcutaneous nodules in rheumatoid arthritis. Of course for those who hold that this disease is nothing more nor less than chronic rheumatism, its association with fibrous growths in the skin is accepted as lending support to their teaching. But, for the most part, those who have been prominent in asserting the rheumatic significance of such nodules have also insisted on the existence of an essential difference between true rheumatic disease and chronic rheumatoid arthritis. Hence the detection of such nodules in cases of the latter disease free from rheumatic taint has compelled those who hold such a view to modify their original theory and to extend nodule formation as a possibility common both to rheumatic and rheumatoid disease. That subcutaneous fibrous nodules do occur in chronic rheumatoid arthritis is now entirely beyond doubt. It is true that, speaking generally, such nodules are larger and more persistent than those which not infrequently mark the rheumatic attacks of childhood. But both in these and in other respects no sharp line of demarcation can be drawn between the two groups, and, indeed, both in macroscopic and microscopic characters there is a region where the two mingle and overlap. Hence it must be allowed that a pathological process, namely, the formation of fibrous nodules in the integuments which was originally claimed as peculiar to rheumatic disease, does, and that not infrequently, mark the course of cases of rheumatoid arthritis, and therefore, on the supposition that the latter is not a true rheumatic disease, the original thesis regarding the absolute significance of such nodules must be qualified. A search through medical literature affords further ground for emphasising this qualification and for extending its range. Every now and then a case will be found—and their features in other respects vary widely—in which a description is given of the appearance in the skin of nodules which in their general characters closely resemble, if they do not absolutely correspond to, those described as the peculiar mark of the rheumatic process. In at least one of these cases the patient was the subject of secondary syphilis, and was entirely free from arthritis and from all other evidences of rheumatism. This case is recorded by Sir Stephen Mackenzie,[2] and its significance is confirmed by a somewhat similar experience noted by Sir Dyce Duckworth,[3] in which also no rheumatism was present. Bannatyne[4] has seen nodules in one case of gonorrhœal arthritis, and he quotes syphilis, influenza, and Malta fever among their possible causes. In connection with the last-mentioned, an observation of much interest has recently been published by Mr. Cantlie.[5] The patient was a man who had been living in Southern India, and had observed a number of small shot-like nodules in the skin of his limbs. Each lasted about three weeks, and then disappeared. The possibility of the nodules having a filarial origin was disposed of by an examination of the blood, and the microscope showed a connective tissue structure. Mr. Cantlie suggests that the condition was due to an evanescent obstruction of the lymphatics. It is to be noted that in this instance the development of each nodule was associated with redness and some irritation of the skin, and on these grounds alone it may be doubted whether the nodules have the same direct etiological relationship as those caused by rheumatism. These latter, as is well known, do not cause redness of the skin, nor are they tender unless they have been irritated by friction or otherwise. All the same their evanescent character suggests some temporarily acting agent producing its results through the medium of the blood or lymph circulation. In conclusion, it may be again pointed out that, in spite of such exceptions and modifications as have here been described, the clinical position in relation to subcutaneous fibrous nodules remains without material modification. As exceptional curiosities they have been found to be connected with various diseases, but their abiding practical value is as evidence of the rheumatic process. Further, in relation to prognosis, it must still be allowed that their common association is with the graver and more progressive forms of pericarditis and endocarditis. Here, also, occasional exceptions have been quoted, as, for example, in a case by Dr. Hugh Thursfield[6] in which nodules were present, together with periostitis and slight effusion into one knee-joint, but without endocarditis. Such an instance, however, has no great force unless freedom from cardiac disease can be recorded over a number of years. This was well shown in a discussion of Dr. Thursfield's case where Dr. Voelcker referred to a patient who had been shown to the Clinical Society as an example of subcutaneous fibrous nodules apart from heart disease, and was afterwards admitted to hospital with an attack of rheumatism and definite endocarditis. In short, the diagnostic value and prognostic significance of such nodules, save in the case of rheumatoid arthritis, remain much as they were defined by Barlow and Warner in the paper they communicated to the International Medical Congress in the year 1881.

[1] Transactions of the Society for the Study of Disease in Children, Vol. I. [2] Clin. Soc. Trans., Vol. XVI., p. 188. [3] Ibid., p. 190. [4] Lancet, Feb. 23, 1901. [5] Brit. Med. Jour., Sept. 17, 1904. [6] Clin. Soc. Trans., 1901.

